# Qualitative analysis of activated sludge using FT-IR technique

**DOI:** 10.1007/s11696-018-0514-7

**Published:** 2018-06-09

**Authors:** Michał Kowalski, Katarzyna Kowalska, Jarosław Wiszniowski, Jolanta Turek-Szytow

**Affiliations:** 1 Faculty of Energy and Environmental Engineering, Department of Air Protection, Silesian University of Technology, 22B Konarskiego Str., 44-100 Gliwice, Poland; 2 Institute of Epidemiology, Helmholtz Zentrum München, German Research Center for Environmental Health, 1 Ingolstädter Landstr., 85764 Neuherberg, Germany; 3 Faculty of Energy and Environmental Engineering, Environmental Biotechnology Department, Silesian University of Technology, 2 Akademicka Str., 44-100 Gliwice, Poland; 4 The Biotechnology Center, Silesian University of Technology, 8 Bolesława Krzywoustego Str., 44-100 Gliwice, Poland

**Keywords:** FT-IR, Spectral analysis, Wastewater treatment, Activated sludge, Petroleum compounds

## Abstract

The ability to measure and control the composition of activated sludge is an important issue, aiming at evaluating the effectiveness of changes occurring in the sludge, what determines its usefulness to treat wastewater. In this research, diffuse reflectance infrared Fourier transform (FTIR–DRIFT) technique was used, which relies on measuring the reflectance of the powdered substance’s surface layer and capturing spectra in range of infrared wave. First, spectra correlation table of the substances mostly occurring in wastewater was developed to assess the main components of the tested samples of activated sludge. The simplest compounds containing functional groups characteristic for particular chemical classes were chosen: peptides (peptone and albumin), fats (glycerin and fatty acids), carbohydrates (glucose and sucrose), nitrogen compounds (NaNO_3_ and NH_4_SO_4_), sulfur compounds (Na_2_SO_4_ and Na_2_S_2_O_3_), silicate, etc. The spectra of those substances were captured and characteristic absorption bands for respective bonds in the function groups were assigned. Second, samples of activated sludge from lab-scale membrane bioreactors (MBRs), which purifies petroleum wastewater, were taken. Samples were properly prepared (lyophilization and homogenization) and their spectra were captured. During spectra analysis, previously developed correlation table was used. In obtained spectra of activated sludge, absorption bonds characteristic for amides, peptides, carbohydrates, fats, and aliphatic was identified. The spectra profile of the sludge sample from MBR feed with petroleum wastewater was slightly different from the control MBR sample’s spectra. Intensity of bands in the area characteristic for aliphatic compounds and phenols was clearly higher. This study proves the usefulness of FT-IR technique to observe changes in the chemical composition of activated sludge.

## Introduction

The ability to measure and control the composition of the activated sludge is an important issue, aiming at assessing the effectiveness of the changes taking place in sediment to evaluate its usefulness in the wastewater treatment process. The previous studies conducted in Western countries, the European Union, the USA, and in Asian countries point to use for this purpose techniques of Fourier transform infra-red (FT-IR) (Jiao et al. 2010; Kim et al. 2013; Reed et al. 2011; Zeng et al. 2016).

This technique is widely used for determination of composition in different media, such as water, soil, or plants. For example, it could be used to assess the changes in soil organic matter induced by irrigation with treated wastewater (Bernier et al. 2013; Parolo et al. 2017). Another application of FT-IR could be determination of metabolic profiles of *Ulva lactuca* plant, after exposure to oil diesel and gasoline (Pilatti et al. 2017).

This method is based on capturing spectra of the chemicals in the range of infrared wavelengths and allows determining the absorption bands of the substances, and thus identifying them. However, the effectiveness of the quantitative method determines the type of the chosen transformation method of the obtained spectrum during further analysis. Simple analysis allows only determining the occurrence of chemical compounds. More sensitive methods lead to compare the intensity of absorption bonds and specific spectra transformation gives an opportunity to quasi-quantitative estimation of chemical concentration.

In recent years, growing interest in the possibility of the use near infrared range (NIR) for diagnostic purposes in real time is observed. This method provides an opportunity to continuously monitor the sludge used in the process, or the composition of the influent to the waste water treatment plant (WWTP) as an early warning system. Specific probes mounted directly in the influent collectors of WWTP and coupled with the proper software could be a source of important information for the WWTP control. This method is non-invasive, fast, and relatively cheap, does not require chemicals, and does not produce any chemical waste. However, authors admit that the technique requires refinement and proper calibration, due to its sensitivity to changes in physical parameters such as temperature, degree of aeration, movement, or light scattering (Dias et al. 2008; Sarraguça et al. 2009).

Another method is the FTIR–attenuated total reflection (FTIR–ATR)—method of recording attenuated total reflection technique, which allows to the quantitative determination (directly on the membrane) of proteins responsible for the phenomenon of fouling (Delaunay et al. 2008; Pereira and Yarwood 1996). It requires special membrane material like containing in its structure polyether sulfone (PES) or polysulfone (PSU). Authors conclude that this method is useful to study the phenomenon of fouling in real time. The FTIR–ATR technique is also used to detection of changes occurring in the biofilm (Quilès et al. 2010). Research carried out concerning usage of *Pseudomonas fluorescens* shows that it is possible to use this technique to continuously monitor changes in metabolism starting from the early stage of the biofilm formation. Resulting spectra differ greatly among themselves, showing the different paths of development of the biofilm.

The aim of this study was to develop a rapid and reliable method for the evaluation of the activated sludge composition, using FT-IR techniques, and also identifying its main components.

## Experimental

### Sample fixation

In the first stage of the experiment, the spectra library of the compounds mostly occurring in municipal and coking plant wastewater was developed. The simplest chemical compounds, containing characteristic function groups for the specific classes of chemicals, were chosen. From the available reactants, only those with the highest purity were taken to the further analysis. Usually, spectra of the specific compounds were captured in three different concentrations (w/w concentration, relative to KBr, and optically transparent diluent) to assess the increase of intensity of absorption bands within concentration. With some of them, due to problems with dosage substances in liquid phase, spectra were captured in 1–2 concentrations. The list of model compounds with their purity and concentration is presented in Table 1.

**Table 1:** Model compounds used in the study, their purity, and concentration

Compound	Purity	Conc. 1 (%)	Conc. 2 (%)	Conc. 3 (%)
Acidic sodium carbonate [NaHCO_3_]	–	3	5	10
Albumin	–	1	5	10
Ammonium sulphate [NH_4_SO_4_]	PA	1	5	10
Dry meat extract	–	1	5	10
Glucose [C_6_H_12_O_6_]	PA	1	5	10
Glycerol [C_3_H_5_(OH)_3_]	Pure	5	7	–
Lactose [C_12_H_22_O_11_]	PA	1	5	10
Oleic acid [C_17_H_33_COOH]	Pure	3	6	–
P-30^a^ (petroleum compound)	–	5	–	–
Peptone G	–	1	5	10
Phenol	Pure	1	5	10
Potassium cyanide [KCN]	Pure	1	5	10
Potassium thiocyanate [KSCN]	PA	1	5	10
Silicate [SiO_4_]	Pure	1	5	10
Sodium carbonate [Na_2_CO_3_]	Pure	3	5	10
Sodium nitrate [NaNO_3_]	PA	1	5	10
Sodium nitrite [NaNO_2_]	PA	1	5	10
Sodium sulphate [Na_2_SO_4_]	Pure	1	5	10
Sodium sulphite [Na_2_SO_3_]	PA	1	5	10
Sodium thiosulfate (dry) [Na_2_S_2_O_3_]	PA	5	–	–
Sodium thiosulfate (liquid) [Na_2_S_2_O_3_]	PA	5	–	–
Sucrose [C_12_H_22_O_11_]	PA	1	5	10
Tween (detergent)	Pure	4	6	–

^a^P-30 was vacuum distillate of crude oil (boiling point 135–402 °C) furnished by PKN Orlen oil refinery (Poland) (Wiszniowski et al. 2011)

Every sample was put into dryer set to 105 °C and dried for at least 6 h to remove water. According to the adopted methodology, FTIR–diffuse reflectance infrared Fourier transform (FTIR–DRIFT) technique was used. Method was based on the instruction given by the producer of the instrument for the spectra capturing (PIKE Technologies 2009). Each dried sample was rubbed in agate mortar. Then weighted out the specific mass and added powdered, dried potassium bromide (Spectroscopic grade KBr Graseby, Specac, UK) to obtain proper w/w concentration sample diluent. According to the methodology, to assess the changes in the intensity of absorption bonds of specific functional groups, chosen samples were prepared in concentrations 1–10% (w/w). Then, the DRIFT pellets were prepared to obtain homogeneous mixture.

### Spectra capturing

Previously prepared pellets were put into the special holder (Easidiff diffuse, PIKE Technologies, USA) and placed in the Fourier transform infrared spectrophotometer BIO-RAD FTS 135 (BIO-RAD, USA), fitted with DTGS detector (Denaturated Trigliceryn Sulphate). Spectra were captured in mid-IR range 4000–400 cm^−1^, scanning resolution 4 cm^−1^, 256 single scans, and initial delay 300 s (for each sample). For the further analysis, Win-IR (BIO-RAD, USA) application was used. Obtained, averaged single beams for each sample were transformed into Kubelka–Munk (K–M) format, using as a background clear KBr (individual for each set of analysis). This method allows to indirectly assess the concentration, according to Eq. (1), where *k*—constant, *C*—concentration, *K*—adsorption coefficient, *S*—diffraction coefficient, and *R*—reflectance, and is preferred to samples weakly absorbing radiation (Džimbeg-Malčić et al. 2011; Hunter Lab 2008):

1 $$kC = \frac{K}{S} = \frac{{\left[ {1 - R^{2} } \right]}}{2R}.$$

Obtained spectra were prepared for the further analysis. Some of them were smoothed using Sav–Golay method to enable identification of the occurring absorption peaks, without losing diagnostic information.

### Preparation of environmental samples

During the next stage of the experiment, activated sludge from the lab-scale system consisting of twin, separate membrane bioreactors (MBRs) was sampled. Activated sludge used as an inoculum originated from municipal wastewater plant in Zabrze (Poland). One MBR was conducting the process of petroleum-contaminated wastewater treatment (MBR B), while the other (MBR A) was considered as a control. The experiment was lasting 122 days and was divided into five stages, according to increasing doses of P-30 (0–1500 µL/L). System was feed with synthetic wastewater, and medium for MBR B was enriched with dosing petroleum compound (P-30). P-30 is distillate of crude oil with boiling point 135–402 °C, mostly consisting of poorly degraded aliphatic and aromatic hydrocarbons. Own research revealed the content of PAH, i.e., naphthalene, phenanthrene, anthracene, fluoranthene, and pyrene. The composition of P-30 fraction varies depending on the course of distillation. It is used as an addition to many industrial products, i.e., paints, impregnants, lubricants, etc. Activated sludge was collected from MBR A and MBR B approximately every 3 weeks.

Each time sampled 50 cm^3^ of activated sludge from MBR A and MBR B. Then, filtered it and the residual was put into clear boxes and frozen. After this, activated sludge’s samples were lyophilized in device Alpha 1-2 LD (Martin Christ GmbH, Germany), for 5 days in vacuum 0.012 mbar. After that, samples were rubbed with dried KBr in ratio 15:300 (w/w), according to previously prepared model samples. For each sample, prepared three pellets consisting of KBr sample in the same ratio and analyzed using Win-IR application. After sampling, the replicates spectra for each sample were averaged.

## Results and discussion

### Spectra library

Based on captured spectra of the model compounds, and also their chemical structure (Mazurkiewicz and Salwińska 2000; Cygański 2002), comparing obtained results to reference correlation tables (Pretsch et al. 2000), spectra library was developed. It consists of obtained data of absorption bands of substances mostly occurring in wastewater. Library is shown in Table 2.

**Table 2:** Library for analysis of the activated sludge composition

Wave number (cm^−1^)	Shape	Intensity	Bond	Compound(s)	Class
3650-3200	b	str	st O–H	Glycerol	Polyalcohol
	b	m		Glucose	Monosaccharide
	b	m		Lactose	Sacharide
	b b	m m		Sucrose Phenol	Saccharide phenols
3300-2800	b	m	st NH_3_ (N^+^–H)	Albumin	Protein
	b	m		Ammonium sulphate	Ammonium
	b	m		Dry meat extract	Peptide
	b	m		Peptone G	Peptide
3000-2840	s (m)	str	st C–H	Glycerol	Polyalcohol
	s (m)	m		Glucose	Monosaccharide
	s (m)	m		Oleic acid	Fats
	s (m)	w		Sucrose	Sacharide
2900	s	w		Lactose	Sacharide
2360	s	w	st NH^+^	Ammonium sulphate	Ammonium
2075	s	str	C≡N	Potassium cyanide	Cyanides
2045	s	str	SC≡N	Potassium thiocyanate	Thiocyanides
1775	s	w	st C=O	Sodium carbonate	Carbonates
1710	s	str		Oleic acid	Fats
1680	s	str	st C=C	Phenol	Phenols
1655	s	str	st as COO^−^	Albumin	Protein
1650-1575	s (m)	str		Dry meat extract	Peptide
1600	s	str		Peptone G	Peptide
1570	s	w	def NH_2_	Ammonium sulphate	Ammonium
1535	s	str	sy ${\text{NH}}_{3}^{ + }$	Albumin	Protein
	s	str		Peptone G	Peptide
1515	s	str		Dry meat extract	Peptide
1460	s	str	st C=O	Sodium carbonate	Carbonates
	s	str		Acid sodium carbonate	Carbonates
1445-1350	s	m	def C–H (skelet)	Glucose	Monosaccharide
	s	m		Lactose	Sacharide
	s	m		Sucrose	Sacharide
1440	s	m	def CH_2_	Oleic acid	Fats
1410	s	str	CH_2_	Glycerol	Polyalcohol
1400	s	str	S(=O)_2_ (SO_4_)	Ammonium sulphate	Ammonium
1400	s	str	def NH_3_	Dry meat extract	Peptide
1380	s	str	N=O	Sodium nitrate	Nitrate
1270	s	s	st NO_2_	Sodium nitrite	Nitrite
1255	s	m	def NH_3_	Peptone G	Peptide
1245	s	m		Albumin	Protein
1235	s	m	def C=O	Oleic acid	Fats
1135	s	s	st S=O (R–SO–OR)	Sodium sulphate	Sulphate
	s	s		Sodium sulphite	Sulphite
	s	s		Sodium thiosulphate	Thiosulphate
1100-1040	s (m)	s	st C–O	Glycerol	Polyalcohol
1100	s	s	st Si–O	Sodium silicate	Silicate
1100-1000	s (m)	s	st CO	Lactose	Sacharide
	s (m)	s		Sucrose	Sacharide
1075	s	w	S=O	Ammonium sulphate	Ammonium
1015	s	s	C–O (CH_2_–OH)	Glucose	Monosaccharide

*b* broad band, *s* sharp band, *s (m)* multiple sharp band, *str* strong intensity band, *m* medium intensity band, *w* weak intensity band, *st* stretching vibration, *st as* stretching asymmetric vibration, *def* deforming vibration, *sy* symmetrical vibration, *skelet* skeletal vibration

Wave numbers of the peaks corresponding with absorption bands and their characteristic shown in Table 2 were used during analyzing environmental sample’s spectra. In case of developed spectra library, other publications concerning FT-IR analysis, using similar methodology, obtained roughly the same results, and peaks were observed in very similar regions (Quilès et al. 2010; Gulnaz et al. 2006; Wharfe et al. 2010; Cheftez et al. 2006; Amir et al. 2010; Kang et al. 2007; Guibaud et al. 2003).

### Lab-scale samples analysis

During analysis of the activated sludge’s spectra, observed characteristic infrared absorption bands. First of them was broad, mid-intense band between 3700 and 3300 cm^−1^. Clearly visible are maxims at 3300 cm^−1^, stretching vibration O–H of hydroxyl group compounds (polyalcohol and saccharides), and at 3100 cm^−1^, N–H stretching vibration (proteins, peptides). In case of the samples from control MBR A, the relative intensity is increasing according to the duration of the experiment; it may reflect the adsorption of those compounds on the sludge. Whereas spectra obtained from MBR B (treating petroleum wastewater) reflect that samples from the beginning of the experiment have the strongest intensity at 3300 cm^−1^. This may suggest that petroleum compounds affect the adsorption capacity of the sludge.

Observed on 2950 cm^−1^ medium intensity, duplet band reflects alkyl chains (polyalcohols, saccharides, and fats), referred to stretching vibration of C–H bond. Intensity of peaks in this region is increasing with duration of the experiment, especially with MBR B, which may be explained by adsorption of petroleum compounds.

In case of MBR B spectra also observed mid intensity peaks on 1700 cm^−1^, which reflect stretching C=O vibration in petroleum compounds: aliphatic ketones—1695–1730 cm^−1^; aromatic aldehydes—1690–1720 cm^−1^; aliphatic–aromatic ketones—1675–1700 cm^−1^; and amides—1630–1700 cm^−1^. In some samples, this region is masked by other, much more intense bands.

In region 1650 cm^−1^, the mostly intense absorption band in the whole spectra is observed. This signal reflects stretching, asymmetrical vibrations of COO^−^ and is characteristic for peptides and proteins.

Next band occurs in 1535 cm^−1^. It is a sharp, intense peak reflecting corresponding symmetrical vibrations of NH^+^ bond, characteristic for proteins. Relative intensity of this band is increasing with time of sampling. There can also be observed absorption bands near 1450 cm^−1^: monosaccharides (deforming C–H bond vibration), ring, aromatic (mid-intense, 1465–1430 cm^−1^), alkenes (C–H functional group), and amides (N–H group).

In wave number 1245 cm^−1^, region of deforming vibration of NH^+^, absorption band is also observed. This reflects also peptides and proteins and increase of the intensity may be explained by adsorption of them on surface of the activated sludge or indicating the secretion of the extracellular polymeric substances.

The last analyzed region is 1060 cm^−1^, characteristic for C–O bond stretching vibration in glycerol. Occurrence of this peak is an evidence of fats and fatty acids presents in the sample.

**Fig. 1:**
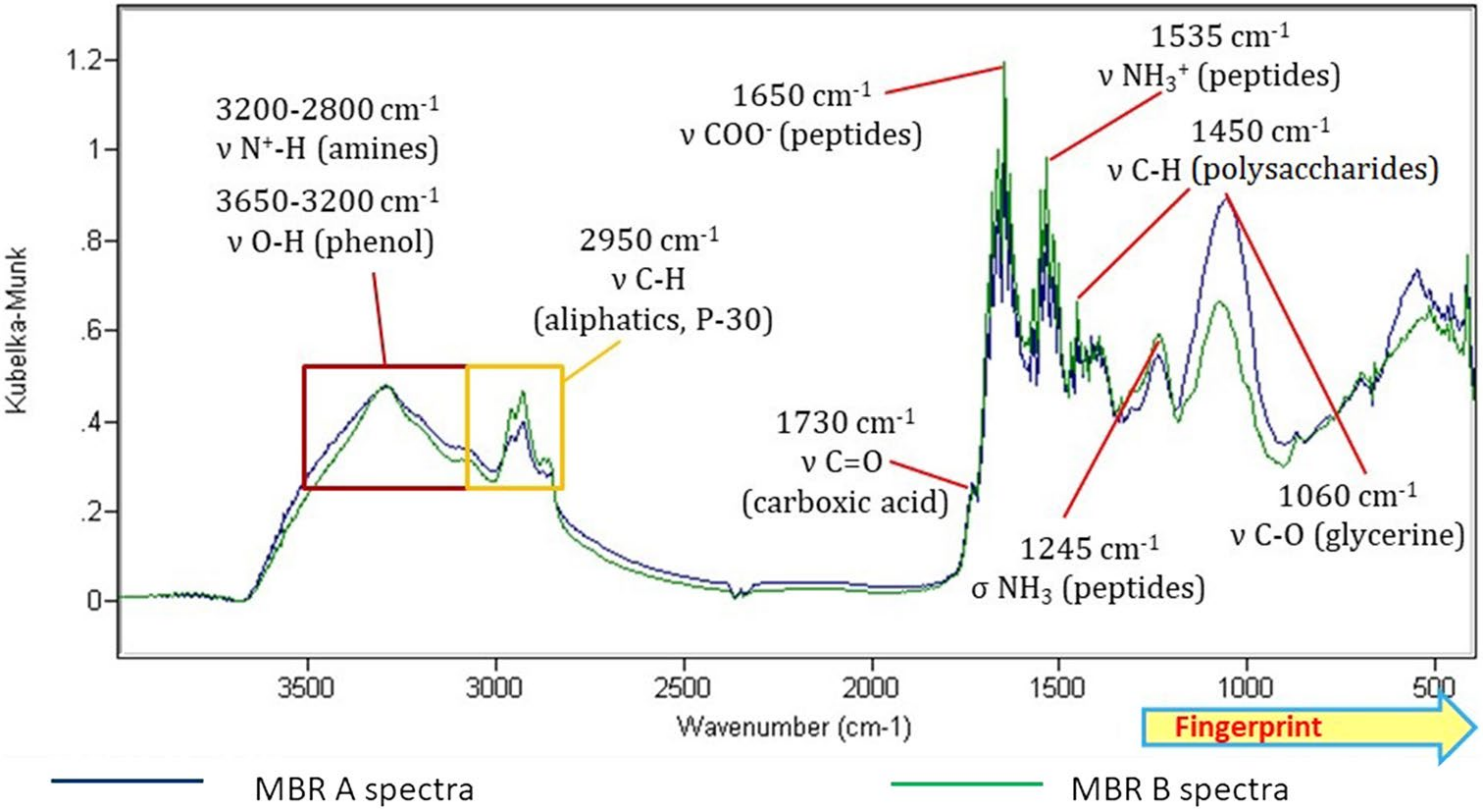
Spectra profile obtained from MBR A (control) and MBR B (working reactor) samples from the late stage of the experiment

Figure 1 shows spectra profile obtained from MBR A (control) and MBR B (working reactor) samples from the final of the experiment.

Summing up, both samples (obtained from MBR A and MBR B) contained peptides and proteins, carbohydrates, fats, and aliphatics. As a reason for the analogies stated that both reactors were inoculated with the same activated sludge (municipal WWTP in Zabrze). Moreover, the only difference in the fed for the reactors was dosing petroleum compounds to the MBR B broth. Differences are visible not so much in position of the absorption bands, but in their relative intensity (according to the methodology, Kubelka–Munk method allows to indirectly assess the changes in concentration by comparing the intensity). It can be concluded that dosing petroleum compounds to the reactor’s feed cause occurring changes in FT-IR spectra of the activated sludge, especially in regions 2950 cm^−1^—aliphatics; 1700 cm^−1^ C=O bond region and 1060 cm^−1^. Due to used transformation methods (Kubelka–Munk), those intensity changes can be quasi-quantitatively assessed. Higher peak in the region 2950 cm^−1^ clearly reflects adsorption of aliphatic compounds on the activated sludge.

The results of FT-IR analysis of lyophilized activated sludge suggest that the presence of characteristic absorption peaks of peptides, lipids, polymeric substances, and carboxylic acids may be referred to presence of functional groups in the microorganism’s organelles and EPS (Extracellular Polymeric Substances) (Gulnaz et al. 2006; Liu and Fang, 2002).

Similar experiment concerning analysis of the FT-IR spectra of activated sludge treating wastewater containing phenols, the appearance of absorption peaks in range 2124-2082 cm^−1^ was observed (Wharfe et al. 2010). The intense of the peak was increasing during the study period, what may be result of adsorption of phenol and/or its metabolites on the surface of flocks. Moreover, the absorption peaks in range 1754-1710 cm^−1^ were observed. Probably, it was connected with vibration of C = O bonds characteristic for carboxylic groups and metabolism of phenol by activated sludge with cleavage of benzene ring. The presence of phenol in wastewater might influence the chemical composition of activated sludge. Other studies concerning investigation of marine plant exposed to petrol and diesel changes in metabolic profile revealed that there were visible changes in FT-IR spectra of plants exposed and non-exposed to xenobiotics (Pilatti et al. 2017).

Studies concerned the comparison of biomass collected from different types of wastewater-treating systems (fixed-film activated sludge, moving bed biofilm reactor, and membrane bioreactor), defined as effectiveness of acid extractable fraction removal; measuring was performed also using FT-IR technique. Revealed that there are differences between specific treating systems, depending on attached biomass (Huang et al. 2017). Another experiment concluded that there are notable variations in relative intensities of several characteristic absorption bands of functional groups among different treated wastewaters, and FT-IR spectra were comparable to those obtained in recent study (Yang et al. 2015).

In experiments conducted by Cheftez et al. (2006), the IR spectra of fraction showing sorption properties to aromatic hydrocarbons were studied. The tested substances were presenting hydrophobic neutral (HoN) and hydrophobic acid (HoA) features. The neutral compounds showed higher sorption properties than the acid compounds. Spectra of both substances differ by the presence of intense absorption peak wavenumbers 1720 and 1650 cm^−1^ in the HoA spectrum and its absence in the HoN spectrum. The range of wavenumber is characteristic to vibration of C=O bonds, what may be referred to the presence of carboxyl group COOH. In the other ranges of frequency, spectra are similar in shape. Zhang et al. (2009) revealed that FT-IR analysis may be used to evaluate the degradation of extracellular substances and suspensions (EBOM—extracellular biological organic matter) by cells capable of generating electricity (MFC—microbial fuel cell).

In other studies (Jiang et al. 2010), spectra of four substances were compared. The changes in the intensity of absorption bands as a measure of biodegradation rate were determined. The results show that fractions containing aliphatic fragments, of secondary amides, carbohydrates, and hydrocarbons were biodegradable. However, the compounds containing aromatic components were not hydrolyse and biodegradable by MFC cell system.

Summarizing, the FT-IR technique appears to be useful in monitoring changes in composition in activated sludge and other media, such as soil. Performing FT-IR analysis of samples provides useful indicators to characterize changes in organic matters, without the need of extraction procedures (Bernier et al. 2013). This analytical approach is simple, cheap and efficient in applications aimed to determine changes in investigated media (Pilatti et al. 2017).

## Conclusions

The obtained results found that fast and reliable analysis of the activated sludge chemical character using FT-IR technique is possible.

- Analysis of the activated sludge spectra based on the prepared correlation table is fast and reliable method.
- Analysis conducted using personally developed correlation table provides clear and reliable results, corresponding to the results obtained in similar experiments.
- Activated sludge can be identified for the type of treated wastewater based on the presented methodology, using the FT-IR technique.
- Changes occurring in the chemical structure of the activated sludge treating petroleum wastewater are visible in the obtained spectra, and can be explained using FT-IR analysis.

## Symbols

*k*: Constant
*C*: Concentration
*K*: Adsorption coefficient
*S*: Diffraction coefficient
*R*: Reflectance

## Greek letters

*ν*: Stretching vibration
*σ*: Deforming vibration

## Subscripts

b: Broad band
def: Deforming vibration
DRIFT: Diffuse reflectance infrared Fourier transform
DTGS: Denaturated trigliceryn sulphate
FT-IR: Fourier transform infra-red
m: Medium intensity band
MBR: Membrane bioreactor
PES: Polyether sulfone
PSU: Polysulfone
s: Sharp band
s (m): Multiple sharp band
skelet: Skeletal vibration
st: Stretching vibration
st as: Stretching asymmetric vibration
str: Strong intensity band
sy: Symmetrical vibration
w: Weak intensity band
